# Patient Perceptions of Dietary Therapies in Crohn’s Disease: A Dietitian’s Perspective

**DOI:** 10.1093/crocol/otae012

**Published:** 2024-02-20

**Authors:** Christine L Scarcello

**Affiliations:** Center for Digestive Diseases, Cedars-Sinai Medical Center, Los Angeles, CA, USA

The study by Jatkowska et al. in this edition of *Crohn’s & Colitis 360* analyzed survey responses from 160 adult patients with Crohn’s disease (CD) to gauge their perception of exclusive enteral nutrition (EEN), partial enteral nutrition (PEN), and other food-based dietary therapies. As inquiry into the associations between diet and inflammatory bowel disease (IBD) management continues to grow, several dietary regimens have been and are continuing to be studied as treatments for inducing or maintaining remission in patients. Surveying our patients provides valuable knowledge of their perceptions of these dietary therapies, and this study was able to bring forth new insights that can be used in clinical practice.

Prior studies on patients’ dietary beliefs in IBD have shown that a majority of patients believe diet to be an important factor. Jatkowska et al. found that 54% of their survey respondents believed diet could help with disease management and 13% had already used dietary therapy to manage CD.^1^ Over half (59%) of 294 adult IBD patients surveyed in a 2019 study likewise showed that patients view nutrition to be as important as or more important than their medication.^2^ The relationship between diet and IBD is not always correlated positively by patients, as omission of specific foods was used more often (in 76.5% of patients) than the utilization of diet-specific therapies (47.6%) or the inclusion of specific foods (56.7%) to improve symptoms.^2^ A 2016 survey of 400 adult patients with IBD reported that more than half of the respondents modified their diet (56%), avoided favorite foods (66%), and imposed food restrictions (68%) to address IBD symptoms.^3^ Half (50%) of these patients had not received nutrition education or advice from a dietitian or healthcare provider, but 67% expressed interest in receiving nutritional advice.^3^

Jatkowska et al. were seeking to determine if willingness to try dietary therapies was influenced by demographics such as age, employment status, prior use, and eating habits. A 26-item questionnaire of closed-ended and open-ended questions about dietary therapies was sent to adult CD patients who were on biologic medications in a single country’s National Health System. Responses revealed that 42% preferred a food-based approach rather than EEN or PEN, but 51% were willing to try EEN and 79% were willing to try PEN.^1^ Only 27% of respondents believed EEN or PEN could be effective for disease management and 63% were uncertain about its effectiveness.^1^ Statistical analysis of the responses found that one’s willingness to trial dietary therapies did significantly vary depending on age, employment status, eating habits, and prior or current use of EEN.^1^ One interesting note here was that patients who had prior or current EEN or PEN usage (38% of all respondents) were more likely to try EEN and also more likely to believe EEN or PEN would be an effective therapy for CD management. This is similar to findings in a study by Guo et al. in which 61.5% of adult CD patients who had completed a 4-week course of EEN for remission were willing to participate in future EEN interventions for remission if needed.^4^

Two open-ended questions were included in the questionnaire that allowed patients to express their concerns for PEN specifically. From thematic analysis of these responses, Jatkowska et al. elicited 24 different themes of concern for PEN; palatability (25%), satiety/hunger (25%), taste fatigue (14%), and the effect on social life (14%) were the most predominant concerns.^1^

The results of this study are beneficial to our clinical practice. Trials of EEN in the literature have already shown that adult adherence is low, and this survey highlights that a significant majority of the patients surveyed were willing to try PEN and food-based dietary therapy as an alternative. There is limited and conflicting evidence at this time and no clear guidelines for PEN or food-based diets as remission-inducing treatment, but there have been studies observing effective PEN and dietary therapy use in adults. One prior study comparing an 8-week EEN regimen to a novel combined EEN/PEN regimen in young adults with CD (aged 16–40) found similar endpoint outcomes in both regimens.^5^ Another study found that PEN when combined with the Crohn’s Disease Exclusion Diet (CDED) showed clinical response in 90% of the children and young adults in the study as well as remission in 62%.^6^ In that study, 4 of the 21 patients chose not to do PEN and elected for CDED alone, and 3 of those 4 had clinical remission by the end of the study.^6^ More research is needed on both PEN and food-based dietary therapies.

Assessing a patient’s individual dietary habits, preferences, and willingness to try certain treatment options are important first steps in ensuring successful implementation of and adherence to dietary therapies. Prior discourse has shown the importance of tailored assessments for the implementation of nutrition interventions. The thorough and insightful care pathway developed by Day et al. for assessing, implementing, and monitoring EEN therapy for adults with CD noted that adherence can be optimized through practical considerations of patient preference, accessibility, affordability, and convenience.^7^ The presence of IBD-focused dietitians as part of a multidisciplinary team to monitor patients’ concerns with EEN, PEN, or specialized diets can aid in promoting adherence by attending to these considerations. Dietitians have extensive knowledge of the formulas available for EEN and PEN, programs from which to obtain formulas, and nutrient modifications that patients may request when they are following specific diet regimens. A dietitian can also address issues related to quality of life—such as one’s relationship with food, concerns about weight or nutrition status, and effects on social interactions—that are associated with dietary therapy interventions.

This study is motivating for future research initiatives studying PEN and food-based dietary therapies since patients have made connections between nutrition and their health. It also further emphasizes the role dietitians have in IBD care. This study has some limitations (including a lack of generalizability and being prone to sampling bias), but it was able to provide us with an important analysis of patients’ willingness to try certain therapies based on demographics and give examples of several concerns in the patients’ own words. The study results also show us that there may be nutrition-related treatment knowledge gaps among CD patients that can be optimized through education, and that adherence to dietary therapies can be enhanced with multidisciplinary care.
